# Physiological conditioning by electric field stimulation promotes cardiomyogenic gene expression in human cardiomyocyte progenitor cells

**DOI:** 10.1186/scrt482

**Published:** 2014-08-04

**Authors:** Aida Llucià-Valldeperas, Benjamin Sanchez, Carolina Soler-Botija, Carolina Gálvez-Montón, Santiago Roura, Cristina Prat-Vidal, Isaac Perea-Gil, Javier Rosell-Ferrer, Ramon Bragos, Antoni Bayes-Genis

**Affiliations:** ICREC (Heart Failure and Cardiac Regeneration) Research Program, Head of Cardiology Service, Germans Trias i Pujol University Hospital, Carretera del Canyet s/n, 08916 Badalona (Barcelona), Spain; Department of Neurology, Division of Neuromuscular Diseases, Beth Israel Deaconess Medical Center, Harvard Medical School, Boston, USA; Electronic and Biomedical Instrumentation Group, Departament d’Enginyeria Electrònica, Universitat Politècnica de Catalunya, Barcelona, Spain; Cardiology Service, Germans Trias i Pujol University Hospital, Badalona (Barcelona), Spain; Department of Medicine, Universitat Autònoma de Barcelona, Barcelona, Spain

## Abstract

The optimal cell lineage for cardiac-regeneration approaches remains mysterious. Additionally, electrical stimulation promotes cardiomyogenic differentiation of stimulated cells. Therefore, we hypothesized that electrical conditioning of cardiomyocyte progenitor cells (CMPCs) might enrich their cardiovascular potential. CMPCs were isolated from human adult atrial appendages, characterized, and electrically stimulated for 7 and 14 days. Electrical stimulation modulated CMPCs gene and protein expression, increasing all cardiac markers. *GATA-binding protein 4 (GATA4)* early transcription factor was significantly overexpressed (*P* = 0.008), but also its coactivator *myocyte enhancer factor 2A (MEF2A)* was upregulated (*P* = 0.073) under electrical stimulation. Moreover, important structural proteins and calcium handling-related genes were enhanced. The cardioregeneration capability of CMPCs is improved by electrical field stimulation. Consequently, short-term electrical stimulation should be a valid biophysical approach to modify cardiac progenitor cells toward a cardiogenic phenotype, and can be incorporated into transdifferentiation protocols. Electrostimulated CMPCs may be best-equipped cells for myocardial integration after implantation.

**Electronic supplementary material:**

The online version of this article (doi:10.1186/scrt482) contains supplementary material, which is available to authorized users.

## Introduction

Cardiovascular diseases remain the leading cause of death in Western countries. Alternative strategies beyond current guidelines are actively sought to repair injured cardiac tissue, and stem cell-based therapies provide a promising path toward achieving this goal. In the past decade, progenitors from different origins have been studied for cardiac-regeneration purposes; however, the optimal cell lineage remains elusive. Despite the existence of resident cardiac stem cells, such as human cardiomyocyte progenitor cells (CMPCs), the regenerative capacity of the heart is limited.

The therapeutic potential of CMPCs was outlined in a pivotal report by Smits *et al*. [1], who demonstrated that CMPCs exhibit a certain degree of *in situ* differentiation into cardiomyocytes, smooth muscle cells, and endothelial cells after intramyocardial injection in a postinfarcted model in mice. Cardiomyogenic differentiation has also been promoted in a cardiac-mimetic electrical stimulation model *in vitro* [2]. Accordingly, we hypothesized that the biophysical conditioning of CMPCs by electrical stimuli might enhance their cardiovascular potential and render them (once electrostimulated) fitting candidates for cardiac cell-therapy strategies.

In this study, we reported CMPCs isolation and characterization; we designed an electrostimulation protocol based on 2-millisecond pulses of 25 mV/cm alternating current, and evidenced gene and protein modulations after electric-field stimulation.

## Results

CMPCs were precisely isolated from human adult atrial appendages after the clonogenic method, as previously described [3]. Cell-collection procedure was approved by the local Ethics Committee (Germans Trias i Pujol University Hospital Ethics Committee), and informed consent was obtained from all patients. The study protocol conformed to the principles outlined in the Declaration of Helsinki.

**Figure 1:**
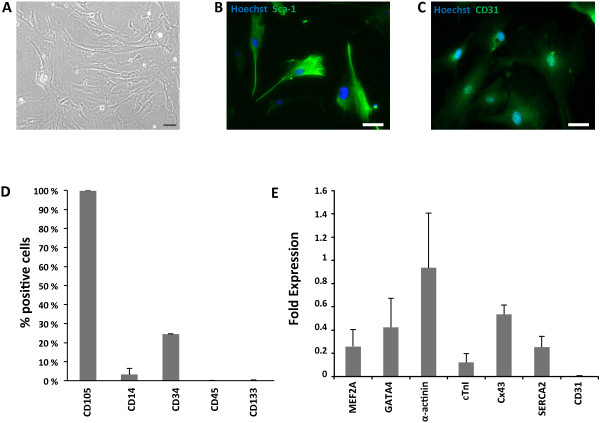
**Baseline characterization of CMPCs. (A)** Bright-field image of CMPCs under standard culture conditions. **(B)** Sca-1 expression (green) in control CMPCs. **(C)** CD31 expression (green) in control CMPCs. **(D)** CMPC surface-marker expression profile under basal conditions. **(E)** Real-time RT-PCR, showing fold expression (2^-ΔCT^) of cardiomyogenic and angiogenic genes in control CMPCs.

Subsequent characterization of CMPCs demonstrated a spindle-shaped, highly proliferating (duplication time: 1.5 ± 0.3 days) population of cells showing high Sca-1 and CD105, low CD34, and undetectable CD14, CD45, and CD133 expressions (Figure 1A-C). A representative CMPC cardiac gene-expression pattern was also found under basal conditions (Figure 1D). In particular, MEF2A and GATA4 transcription factors were expressed, as well as α-actinin, cardiac troponin I (cTnI), the calcium handling-related protein, sarcoplasmic endoplasmic reticulum Ca^2+^ ATPase 2 (SERCA2), and the cell-connection protein, connexin43 (Cx43). Baseline expression of the endothelial marker CD31 was also confirmed in control CMPCs at both gene and protein levels (Figure 1D,E). See Additional file 1 for detailed explanation of materials and methods.

**Figure 2:**
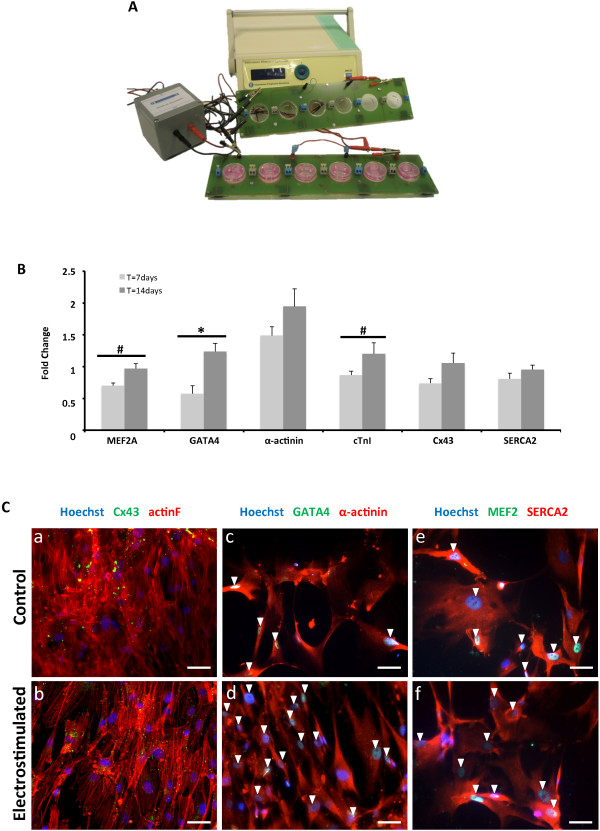
**Genetic and protein analysis after electrical stimulation. (A)** Experimental setup for electric stimulation: the electric stimulator is connected to the printed circuit boards through an isolator stage. **(B)** Real-time RT-PCR of cardiac genes in electrically stimulated CMPCs, as compared with control, at 7 and 14 days. All RT-PCR values were analyzed in duplicate, normalized to GAPDH expression, and shown as fold-change mean ± SEM; *P < 0.05 and #*P* < 0.10, 7-day (*n* = 5) versus 14-day (*n* = 10) stimulation. **(C)** Actin fibers (red) and Cx43 (green), sarcomeric α-actinin (red) and GATA4 (green), and SERCA2 (red) and MEF2 (green) expressions in control **(a, c, e)** and electrostimulated **(b, d, f)** CMPCs, respectively. Nuclei were counterstained with Hoechst 33342. Scale bars = 50 μm. Arrowheads, positive staining of nuclear proteins (MEF2 and GATA4).

Next, CMPCs were electrostimulated by using a custom-made stimulation-unit setup, which comprised a monophasic programmable electrical device, an electrical isolation stage, two printed circuit boards, which enable the fast and robust connection of the electrodes, and a biocompatible polydimethylsiloxane silicone-patterned construct, designed to provide structural support to cells and electrodes (Figure 2A). Although pulses produced by the stimulator are monophasic, the transformer-based isolator creates a negative low-level exponential pulse after each square positive pulse, which ensures zero direct-current average voltage to avoid electrolysis.

The electrical-stimulation protocol consisted of submitting 30,000 seeded cells to 2-ms monophasic square-wave pulses of 25 mV/cm at 1 Hz (alternating current) for 7 and 14 days [4].

Electrical stimulation modulated CMPC gene and protein expression (Figure 2B,C). Figure 2B shows the fold change of the studied cardiac markers at 7 and 14 days. All cardiac markers increased their expression after 14 days of stimulation.

A statistically significant overexpression of GATA4 was observed (*P* = 0.008), but also MEF2A (*P* = 0.073) was upregulated under electrical stimulation. MEF2 proteins are recruited through their DNA-binding domains by the early transcription factor GATA4 to activate cardiac promoters. Both transcription factors are expressed in the developing heart and have similar genetic expression patterns after electrical stimulation.

The presence of early transcription factors might conceivably enhance cardiac protein expression to achieve further a cardiomyocyte-like phenotype (for example, gap junctions for electrical coupling, and sarcomeric proteins for mechanical contraction) (Figure 2Ca-f). Cx43 proteins form gap junctions, which are key elements for impulse propagation throughout the heart syncytium. Cx43 protein was observed in the cytoplasm, as well as at the plasma membrane, particularly in stimulated cells (Figure 2Ca,b), in which its expression was improved. Main structural proteins for the contractile apparatus, such as cTnI and α–actinin, were also augmented (*P* = 0.093 and *P* > 0.1, respectively), although they do not show a striated pattern (Figure 2Cc,d). The absence of sarcomeres could suggest an early stage in the cardiomyogenic differentiation [5].

Additionally, SERCA2 protein was expressed in both conditions (Figure 2Ce,f), and was also slightly enhanced after 14 days of electrical stimulation (*P* > 0.1). SERCA2 proteins are intracellular pumps, which are located in the sarcoplasmic or endoplasmic reticula of muscle cells that are involved in the regulation of the cardiac contraction/relaxation cycle.

## Conclusions

In sum, these data demonstrate that electric-field stimulation of CMPCs enhances cardiac gene expression. Gene modulation is translated to the protein level to promote CMPC phenotype differentiation. Short-term electrical stimulation appears to be a valid biophysical method to modify cardiac progenitor cells toward a cardiogenic phenotype, and can be included in transdifferentiation protocols. Electrostimulated CMPCs may be best-equipped for myocardial integration after transplantation.

## Electronic supplementary material

Additional file 1: **Detailed description of materials and methods used in this study in the additional file.** (PDF 166 KB)

## Abbreviations

CMPCs: Cardiomyocyte progenitor cells
cTnI: cardiac troponin I
Cx43: connexin 43
GATA4: GATA-binding protein 4
MEF2: myocyte enhancer factor 2
SERCA2: sarcoplasmic endoplasmic reticulum Ca^2+^ ATPase 2.
