# Supplementary material for: Physiological conditioning by electric field stimulation promotes cardiomyogenic gene expression in human cardiomyocyte progenitor cells
Source: Stem Cell Res Ther. 2014 Aug 4;5(4):93. doi: 10.1186/scrt482 (PMC4282148; doi:10.1186/scrt482)
Supplement: Supplementary file 1 — Additional file 1: Detailed description of materials and methods used in this study in the additional file. (PDF 166 KB) [file 13287_2014_388_MOESM1_ESM.pdf]

Cell isolation and culture

CMPCs were processed from human adult atrial appendages and cultured as previously described [1]. Briefly, samples were rinsed with cold M-buffer, cut into small clumps removing any fat or scar tissue; then, cells were washed twice in cold PBS and isolated by collagenase A digestion. Thereafter, the cell solution was ground through the 40μm cell strainer on ice, centrifuged and resuspended in PBS. Then, we let the cardiomyocytes sank to the bottom, collected the supernatant in a new 15-mL tube, centrifuged, resuspended and diluted the cells to a concentration of 5 cells/mL in growth medium, and seeded 100μL on a 0.1% gelatin coated 96-well plate with growth medium plus bFGF. The growth medium was composed of EGM-2+M199 (1:3), 10% FBS, 1%Penicillin/streptomycin, 1% α-MEM non-essential aminoacids and 5μg/mL plasmocyn, and it was used for the whole experimentation. Cell collection procedure was approved by the local Ethics Committee (Germans Trias i Pujol University Hospital Ethics Committee), and informed consent was obtained from all patients. The study protocol conformed to the principles outlined in the Declaration of Helsinki.

Electrical stimulation setup

The biocompatible polydimethylsiloxane (PDMS; Sylgard, 184, Dow Corning Corp.) silicone constructs were built using a custom mould that was designed with commercial CAD software (Solidworks), fabricated with poly(methyl methacrylate) (PMMA), and constructed by the assembly of three different layers. The cell pool surface area was imprinted with a regular pattern perpendicular to the direction of the electric field to support cell alignment [2]. The PDMS structure also had dedicated rooms to accommodate the stimulation electrodes. These electrodes were made of a Teflon core (PTFE) (2 mm height, 1 cm length), which was the structure for the twisted platinum

wire (0.5 mm, 30 cm; PTP201; World Precision Instruments).

The PDMS compounds were made by mixing two liquid components in a ratio of 10 parts base to one part curing agent in a liquid state (10:1, by weight) (Sylgard, 184, Dow Corning Corp.). Air introduced into the mixture during stirring was removed by using a vacuum chamber at 700 mbar for 30 minutes. Then, the mixture was smoothly poured into the PMMA mould and cured in an oven (70°C, 2.5-3h). Once the mixture was cured, the mould was carefully removed. PDMS constructs were sterilised by autoclave prior to cell culturing.

Quantitative real-time PCR

Total RNA was isolated from CMPCs using the AllPrep RNA/Protein Kit (Qiagen). cDNA was synthesized using random hexamers (Qiagen) and the iScript™ One-Step RT-PCR Kit (BioRad Laboratories) according to the manufacturer's protocol. cDNA was preamplified with the TaqMan® PreAmp Master Mix Kit (Applied Biosystems) and then diluted 1:5 with RNAse-free water.

Real-time PCR amplifications were performed with 2.5 µL of cDNA in a final volume of 10 µL, containing 5 µL TaqMan 2× Universal PCR Master Mix, 2 µL RNAse-free water, and 0.5 µL of FAM-labelled primer/probe purchased from Applied Biosystems (Foster City), including glyceraldehyde-3-phosphate dehydrogenase (GAPDH) (Hs99999905_m1), myocyte-specific enhancer factor 2A (MEF2A) (Hs01050409_m1), GATA-binding protein 4 (GATA4) (Hs00171403_m1), α-actinin (Hs00241650_m1), cardiac Troponin I (cTnI) (Hs00165957_m1), connexin43 (Cx43) (Hs00748445_s1), sarco/endoplasmic reticulum Ca^{2+}-ATPase (SERCA2) (Hs00544877_m1) and CD31 (Hs00169777_m1). Data were collected and analysed on the LightCycler® 480 Real-

Time PCR System (Roche); each sample was analysed in duplicate and healthy human myocardium was used as a positive control.

The Livak method ($2^{-\Delta CT}$) and Fold Change (|stimulatedΔCT/controlΔCT|) were used to quantify absolute and relative expressions of each gene between electrostimulated and control samples, using GAPDH as an endogenous reference.

Immunocytofluorescence

Cells were fixed with 10% formalin for 15 minutes, permeabilized, blocked in 10% normal horse serum for 2 hours, and incubated for 1 hour at room temperature with primary antibodies against Sca1-like protein (1:100; Abcam), Cx43 (1:100; Sigma), sarcomeric α-actinin (1:100; Sigma), GATA4 (1:50; R&D), MEF2 (1:25, Santa Cruz Biotech), SERCA2 (1:50; Santa Cruz Biotech), CD31 (1:10; Abcam). Secondary antibodies conjugated with Cy2 and Cy3 (1:200; Jackson ImmunoResearch), and actin fibers were stained with Phalloidin Alexa 568 (1:40; Invitrogen). Nuclei were counterstained with Hoechst 33342 ($1:10^4$; Sigma) and healthy myocardium was used as a positive control. Images were acquired with an Axio Observer Z1 inverted microscope (Zeiss).

Statistics

Relative fold expressions of CMPCs were compared using Student's *T*-test, and the statistical difference was determined for the samples from 5 and 10 separate experiments of 7 and 14 days, respectively. The results are presented as mean±SEM. *P* values < 0.05 were considered significant. All analyses were performed with SPSS (21 version, SPSS, Inc.).

REFERENCES

1. Smits AM, van Vliet P, Metz CH, Korfage T, Sluijter JP, Doevendans PA, Goumans MJ: **Human cardiomyocyte progenitor cells differentiate into functional mature cardiomyocytes: an in vitro model for studying human cardiac physiology and pathophysiology**. *Nat Protoc* 2009; **4**(2):232-43.

2. Llucià-Valldeperas A, Sanchez B, Soler-Botija C, Gálvez-Montón C, Prat-Vidal C, Roura S, Rosell-Ferrer J, Bragos R, Bayes-Genis A:. **Electrical stimulation of cardiac adipose tissue-derived progenitor cells modulates cell phenotype and genetic machinery**. *J Tissue Eng Regen Med* 2013. [E-pub ahead of print], doi: 10.1002/term.1710.
